# Dr. Wiley

**Published:** 1911-08

**Authors:** 


### Dr. Wiley

The attempt to oust Dr. Wiley has, apparently fallen flat. It is understood that he will be, technically, reprimanded for exceeding his authority in making necessary expenditures for carrying on the work of his department but the general sentiment seems to be that his powers should be increased, so that he can exercise similar discretion in the future, without question.

There are many interests in the country which have been seriously hampered in their exploitation of the public, by the Pure Food and Drugs Law, and by Dr. Wiley's aggressiveness in its execution. Political interests also are always on the alert to grasp an important office. The sentiment which has manifested itself so vigorously and so promptly, in Dr. Wiley's behalf, has been very largely independent of party politics; some of the interests under control of his department have merited praise by their warm support, the press representing an intelligent public, has with comparatively few exceptions, shown both that sanitary and hygienic regulations are now appreciated at their true value instead of being viewed with hostility, and—more important than anything else in its ultimate issue—that politics in the old sense is losing strength in the control of government and that efficiency in public office will elicit support.

